# Using Geiger Dosimetry EKO-C Device to Detect Ionizing Radiation Emissions from Building Materials

**DOI:** 10.3390/s21020645

**Published:** 2021-01-18

**Authors:** Maciej Gliniak, Tomasz Dróżdż, Sławomir Kurpaska, Anna Lis

**Affiliations:** Faculty of Production and Power Engineering, University of Agriculture in Krakow, Mickiewicza Av. 21, 31-120 Krakow, Poland; tomasz.drozdz@urk.edu.pl (T.D.); slawomir.kurpaska@cyf-kr.edu.pl (S.K.); anna.lis@urk.edu.pl (A.L.)

**Keywords:** materials, radiation, dosimetry

## Abstract

The purpose of the article is to check and assess what radiation is emitted by particular building materials with the passage of time. The analysis was performed with the EKO-C dosimetry device from Polon-Ekolab. The scope of the work included research on sixteen selected construction materials, divided into five groups. The analysis of the results showed that samples such as bricks (first group) and hollow blocks (second group) emit the highest radiation in the tested objects. When comparing these materials, the highest value was recorded when measuring the ceramic block of 15.76 mSv·yr^−1^. Taking into account the bricks, the highest value of radiation was shown by a full clinker brick, 11.3 mSv·yr^−1^. Insulation materials and finishing boards are two other groups of building materials that have been measured. They are characterised by a low level of radiation. In the case of materials for thermal insulation, the highest condition was demonstrated by graphite polystyrene of 4.463 mSv·yr^−1^, while among finishing boards, the highest value of radiation was recorded for the measurement of gypsum board of 3.76 mSv·yr^−1^. Comparing the obtained test results to the requirements of the Regulation of the Council of Ministers on ionizing radiation dose limits applicable in Poland, it can be noted that the samples examined individually do not pose a radiation risk to humans. When working with all types of samples, the radiation doses are added up. According to the guidelines of the regulation, the total radiation dose does not exceed 50 mSv·yr^−1^ and does not constitute a threat to human health.

## 1. Introduction

Because of the current lifestyle of social life, people spend more than 60% of the day in buildings, which increases their exposure to radiation [1]. The radioactivity of building materials may affect the human body using two routes of exposure—external (exposure to radiation) and internal (inhalation of air containing derivatives of radioactive radium isotopes) [2,3]. The impact of radiation on humans is caused by the decay of radioactive isotopes of radium, thorium, and potassium, and the related emission of photons in the form of gamma radiation [4]. Internal exposure caused by radioactive decay of radon with the emission of alpha radiation is much more dangerous for humans [5]. It is now known that primary raw materials, waste, and their by-products used in the production of building materials may contain large amounts of radionuclides [6,7,8,9,10]. As a result of the negative impact of radiation on human health, it is necessary to determine radiation doses from building materials, especially in the case of using materials derived from waste (e.g., phosphogypsum and coal slags) for their production [7,8,9,10,11,12].

Building materials can emit significant doses of gamma radiation indoors owing to the natural content of radionuclides. In order to develop guidelines for the radiological safety of materials, a database was created containing information on ^238^U, ^232^Th, and ^40^K emissions. For this purpose, several thousand materials from various European Union countries were tested in the field of ionizing radiation emission. On the basis of the conducted research, the suitability of the materials for construction purposes was assessed and guidelines for their use were defined [13,14,15,16].

The ionizing radiation dose in Poland for employees amounts to 50 mSv·yr^−1^, provided that the total radiation dose in the next 5 years does not exceed 100 mSv. Similarly, the radiation dose of 1 mSv·yr^−1^ for the general population may be exceeded, provided that the total radiation dose in the next 5 years does not exceed 5 mSv. The literature on the subject also specifies special cases of exposure to radiation—for instance, a woman, from the moment of notifying the head of the organizational unit about pregnancy, cannot be employed in a position where a newborn child could take a dose greater than 1 mSv [13,14,15,17]. The concentration of radionuclides in buildings depends on the speed at which radon enters the interior, the air-tightness of the rooms, the ventilation method, and the frequency of ventilation. The highest concentration of radon is recorded in the basements and on the ground floor of the buildings. The concentration of this gas varies depending on the season and time of day. Radon contamination in winter is higher than in summer, maximum at night and early morning, and lowest at noon [18].

The construction industry and the building materials market are developing rapidly all over the world. Because of the limited access to specialized laboratories, the control of building materials causes problems with the management of radionuclide emissions in buildings. The current radioactivity control systems for building materials may result in the marketing of materials with an increased level of radiation. Therefore, it is necessary to investigate and determine the possibility of a quick, non-invasive method of determining the doses of ionizing radiation emitted from building materials. The aim of this study is to present the application of a device using a Geiger window counter to evaluate the total dose of ionizing radiation from typical building material.

## 2. Materials

The selection of building materials for research was preceded by an analysis of the available databases on natural radiation emissions for these materials published in scientific journals and Euratom Basic Safety Standards Directive [14]. The most comprehensive list of the natural radioactivity of building materials was presented by Trevisi et al. [19], who analyzed data for more than 8000 samples of building materials used in the European Union.

**Figure 1:**
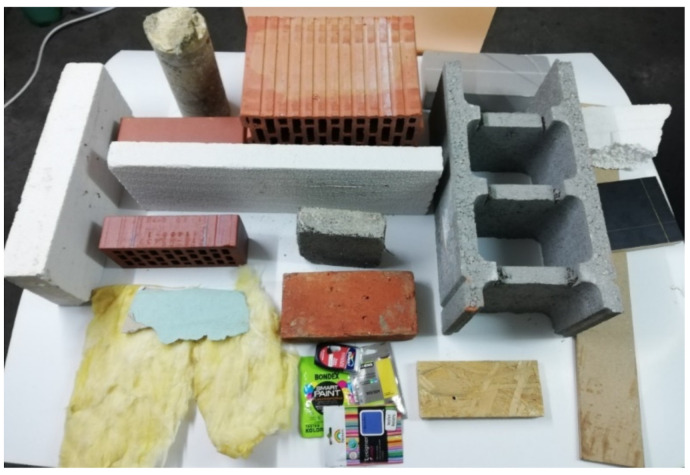
Examples of building materials used in the research.

The research material used for the measurements was typical building materials used in the construction of buildings in Poland. For the research, the 16 most often used building materials were selected, based on information obtained from architectural offices and companies supplying construction sites (Figure 1).

The selected materials were divided into five groups—bricks (construction, full clinker, hollow mould, and classic), hollow bricks and tiles (ceramic, slab, aerated, and shuttering), insulation materials (classic polystyrene, graphite polystyrene, glass wool, and mineral wool), finishing plates (plywood board, gypsum board, Oriented Strand Board (OSB), and MultiFunktionsPlatte (MFP)), and tiles (ceramic and metamorphic). The division of materials into groups was done on the basis of the Euratom Basic Safety Standards Directive [14]. As the reference material, brown soil made from Jurassic limestones collected from the experimental field belonging to the University of Agriculture in Krakow was used. The soil has not been cultivated for at least 15 years.

For the tests, 10 representative samples of each building material were randomly selected from the building materials market in Poland. Each sample was submitted to the testing procedure three times to determine the mean cumulative radiation dose. The cumulative radiation dose I (mSv·yr^−1^) was used to assess the radioactivity of the material [13,14,15,20]. Its purpose is to determine the radioactivity of materials. It is calculated as a weighted average of the concentrations of Radium, Thorium, and Potassium isotopes according to Equation (1):

(1) $$I=\frac{C_{Ra-226}}{300\;\mathrm{Bq}\cdot {\mathrm{kg}}^{-1}}+\frac{C_{Th-232}}{200\;\mathrm{Bq}\cdot {\mathrm{kg}}^{-1}}+\frac{C_{K-40}}{3000\;\mathrm{Bq}\cdot {\mathrm{kg}}^{-1}}$$

where *C_x_*—measured activity concentration (Bq·kg^−1^).

The obtained cumulative dose values are different in different countries around the world. In the European Union, European technical guidance RP112 [13] applies in this respect. This is a reference document published by the European Commission in view of future legislative initiatives. It suggests to base radioactivity controls of building materials on a cumulative dose criteria for control and an exemption level. RP112 proposes that a cumulative dose should be chosen on a national basis in the range 0.3–1 mSv·yr^−1^ of excess whole gamma dose to that received outdoors [13,19,20].

Information on average radioactivity was obtained from available databases presented in the literature. Presented radioactivity emissions was obtained with the use of high-purity germanium (HPGe) gamma-ray spectrometry (Table 1). The low-background γ-spectrometer, with a 10 cm thick lead shield was equipped with a model GC3018 (Canberra) HPGe detector. This system represents 30% efficiency relative to 3 × 3 of NaI crystal and resolution 1.80 keV (FWHM) at 1.33 MeV. GENIE-2000 software was used for peak analysis [19,20].

## 3. Methods

The EKO-C dosimeter by Polon-Ekolab was used to determine the total radiation dose I. The device is based on the G-M counter and detects radioactive contamination and measures their surface activity. The instrument is capable of detecting radioactive sources α, β, γ, and X. The device measures the dose rate γ and X in the range 0.01–1000 μS·h^−1^, surface contamination with α emitters in the range 0.1–10,000 Bq·cm^−2^, and gives the number of α and β counts in the range 0.1 to 10,000 cps. The energy range of the measured ionizing radiation is different for various types of radiation: for α radiation, it is above 4 MeV; for β radiation, it exceeds 100 keV; and for X and γ photon rays, it ranges from 1.5 to 50 MeV. Measurement with the EKO-C radiometer is burdened with the basic error, which is less than 50% for α and β radiation, and 15% for X and γ radiation (with reference to isotope Cs-137). According to the manufacturer of the device, EKO-C can be used to control shields against X and γ radiation, as well as to control surface radioactive contamination with α, β, and γ nuclides. The EKO-C dosimeter is adapted to the determination of radiation dose described in the European Commission guidance document [13,14] in one measurement. The dosimeter allows for faster measurements of radioactivity than standardized methods (e.g., with the use of HPGe (hyperpure germanium) ‘GMX’ gamma ray detectors), and does not require the use of calculations of the registered radiation doses.

**Figure 2:**
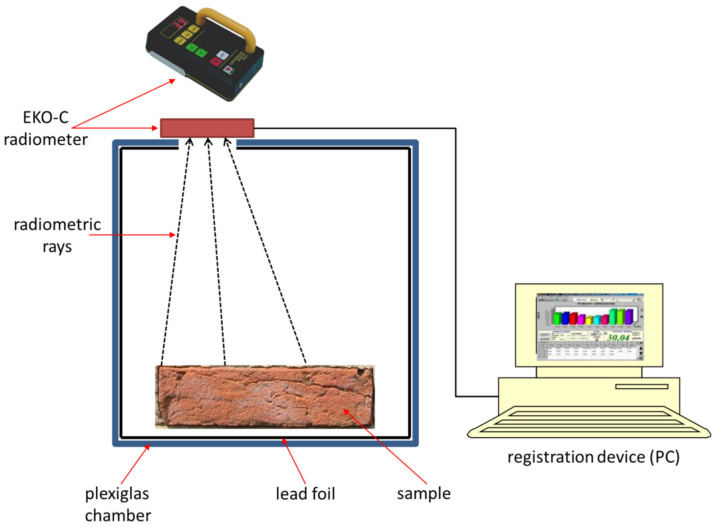
Scheme of the measuring stand.

For measuring the materials shown in Figure 1, a special measuring stand was created—Figure 2. The stand includes a measuring chamber (50 cm × 50 cm × 50 cm) made of plastic with a wall thickness of 10 mm and lined inside with a 4 mm thick lead foil. The measuring chamber has the slit in upper cover for mounting the EKO-C dosimeter.

Before radiation measurement, each sample of the material was reduced to a volume of 100 cm^3^. The total radiation dose was measured by placing each sample of building material in the middle of the bottom cover of the measuring chamber. After the chamber was closed, the dosimeter was turned on and the radiation emission was measured for 5 min. The dosimeter sampling time was 10 s. After the set time had elapsed, the building material was rotated in the chamber to another plane (randomly) in order to repeat the radiation emission measurements. The entire measurement procedure was repeated four times for each material. After the end of the measurement series, the average radiation dose of the material was calculated based on the measurements recorded by the dosimeter. Subsequently, on the basis of the obtained measurements, the expanded uncertainty was calculated in order to determine the significance level of the obtained results—in pursuance of the calculation methodology used in accordance with PN-ISO 5725, ISO 13528:2015 and ILAC-G17. The obtained values were checked by the Dixon test for the Qcrit 0.479 value and using the analysis of variance (ANOVA) for *p* ~ 0.05 and *k* = 2.

## 4. Results

Bricks belong to the first group of construction materials. The research covers the four most popular types of bricks used in construction in Poland (Table 2). The highest value of radiation was recorded for hollow mould bricks (>11.5 mSv·yr^−1^). The lowest maximum radiation dose values are demonstrated for classic and construction bricks (>6.2 mSv·yr^−1^). The highest stability of the obtained results was achieved for solid clinker bricks, and the smallest measurement error for construction bricks. The obtained research results also indicate the negative impact of the use of ashes in the clinker burning process, which is characterised by high radioactivity [6,7,8,9,10]. The obtained research results indicate that full clinker bricks and hollow mold bricks emit twice as much radiation as the reference soil. The conducted studies show that the tested building materials exceed the European standards of cumulative radioactivity more than 10 times [13]. The obtained results are comparable to the results achieved by Trevisi et al. [19] and Gandolfo et al. [21].

The second group of materials includes concrete products from which the skeleton of buildings is constructed. In this group, the analysis covered ceramic and shuttering blocks, aerated concrete, and concrete floor slabs (Table 3). The results of annual cumulative radiation indicate that blocks and concrete products do not differ in radiation doses within the subgroups. The research demonstrated higher radiation from hollow blocks (approximately 30%) than from concrete. As in the case of bricks, concrete also contains ashes, which increase its radioactivity. The conducted research also shows that the emission of radiation is 2–3 times higher than the reference material. Concrete or cement materials in comparison with the group of bricks contain up to 1.5 times more radionuclides. The obtained test results are 3.5 times higher than those described in the studies [19,20,21].

The third group of considered materials are insulation materials—polystyrene and wool (Table 4). There were no significant differences in radiation doses in the polystyrene subgroup. The maximum annual dose of radiation from these materials is 4.99 mSv·yr^−1^. In the subgroup of wools, glass wool (2.71 mSv·yr^−1^) indicates a lower maximum radioactivity. The analysed materials also show that the cumulative radiation dose is approximately two times lower with the use of mineral wool than with polystyrene. The tested materials are characterized by up to 50% lower content of radionuclides compared with the reference sample. The lower concentration of radionuclides than in the case of materials used in the construction of walls and ceilings of buildings results from the use of highly selected materials for their production. These products also contain ash admixtures, which can significantly affect the emission of radiation. The use of the analysed materials in construction does not exceed the standards described in [13,20].

Finishing boards belong to the fourth group of building materials (Table 5). The highest cumulative radiation dose was recorded for gypsum board (4.20 mSv·yr−1). Finishing boards are one of the most important building elements. In many buildings, they are the basic material replacing plasters, which additionally has insulating properties. The most commonly used in construction are gypsum boards, which emit an average of 40% less radiation than the reference soil. These boards are complemented by boards made of wood (OSB, MFP), which, because of the origin of the material, contain traces of radionuclides and emit less radiation. The obtained test results confirm the research carried out by Trevisi et al. [19] and Gandolfo et al. [21]. The use of the analysed materials in construction also does not exceed the standards described in [13].

The last group of building materials constitute ceramic and natural stone tiles (Table 6). Ceramic and stone tiles represent a very specific group of building materials owing to the origin of the raw material used in their production. Tiles made of natural materials with a low radionuclide content (e.g., silica, clays, clay minerals) are used in Poland. The use of selected raw materials translates directly into the radioactivity concentration, which is five times lower than the reference sample and does not exceed 1 mSv·yr^−1^. Comparing the radiation emission of tiles with other construction materials, a significant difference can be seen, reaching even 15 times the average values in comparison with bricks and concrete products. The conducted analyses confirm the results of Trevisi et al. [20] and Gandolfo et al. [21]. These materials also meet the requirements of the RP 112 document [13].

The comparison of the materials used in the research shows that the most radioactive group are hollow bricks owing to their structure. The raw materials used to make blocks or concretes have the most radiation-emitting substances [4,6]. Producers, through legal regulations, must restrict the addition of components such as fly ash, which themselves have a high level of radiation [4,6,12]. Replacing hollow blocks or concrete is a problematic issue as other materials used as substitutes also have an increased level of ionizing radiation. The test samples from the group of bricks constitute materials that can compete with blocks or concrete. Brick, which belongs to building ceramics, also radiates, although not as actively as concrete products and their derivatives. During firing, the radioactive activity of bricks increases [7,9,12,19]. The radiation between the brick and the block varies from 2.26 to 5.25 mSv·yr^−1^. The tested materials, which are intended for thermal insulation, exhibit much less activity. The lowest radiation was recorded when measuring glass and mineral wool. Comparing these samples to all other analysed materials, their radiation is definitely the lowest. Finishing boards constitute mainly wood-based materials and plasterboard panels. Among these materials, plasterboard panel is distinguished by the highest radiation value recorded at the measurement of 3.76 mSv·yr^−1^, and the lowest at the OSB board, of 2.89 mSv·yr^−1^. Comparing all four groups of materials, the blocks are characterised by the highest radiation dose, followed by brick; finishing board; and, finally, insulation materials. Finishing boards constitute the most consistent group, while the greatest differences can be observed in the group of blocks and concrete slabs.

Comparing the obtained test results to the requirements of the Regulation of the Council of Ministers on ionizing radiation dose limits applicable in Poland (Journal of Laws No. 20, item 168 of 2005), it can be noted that the samples examined individually do not pose a radiation risk to humans. When working with all types of samples, the radiation doses are added up. According to the guidelines of the regulation, the total radiation dose does not exceed 50 mSv·yr^−1^ and does not constitute a threat to human health.

In order to determine the actual radiation dose to which the user of the building is exposed, a simulation of the structure of a typical living room was performed, finished with the use of the following materials: graphite polystyrene, concrete, concrete slab, perforated clinker brick, ceramic block, plasterboard, OSB board, ceramic, and stone tiles. The Monte Carlo method was used for the simulation [22]. The conducted simulation showed that, assuming the measured radiation doses (Table 2, Table 3, Table 4, Table 5 and Table 6) and the exposure time of 5000 h·yr^−1^, the effective radiation dose amounts to 0.28 mSv·yr^−1^. The obtained results demonstrated that the combination of materials with different radiation emissions gives a total lower radiation result than considering them individually. The research also confirmed that the combination of materials with differentiated radioactivity meets the requirements of the reference dose of 1 mSv·yr^−1^ described in [23] for indoor and outdoor external irradiation.

## 5. Discussion

The use of radiometric radiation assessment confirms the presence of radionuclides in building materials. Compared with the standard radioactivity assessment method using Gamma-ray spectrometry with HPGe detectors, proportional and stable measurement results were obtained. The emission of ionizing radiation from most materials is fully consistent with the guidelines of Polish law. Based on the conducted experiments, it is concluded that an important element of the reduction of ionizing radiation in construction is the introduction of quality classes of materials. The classification should be constructed on the basis of the indicators that determine the origin and usage—in particular, this applies to bricks and concrete products that emit the most ionizing radiation. Rational use of waste and protection of natural resources belonging to the national guidelines and policy for sustainable development, the production of building materials should be based on the assumption of harmlessness to human health. Manufacturers ought to be involved in testing the radioactivity of materials and should present information on the radioactivity of materials on the packaging. Thanks to such actions, the public will become more aware of the health risks posed by exposure to ionizing radiation. The research results presented in the publication can be a reference for people in the field of trade and use of building materials, thus improving the radiological safety of the environment.

Using the RP112 guidelines [13], the collected results were assessed in terms of radioactivity using the cumulative dose ratio. The obtained research results indicate that the use of index I may lead to limitations in the use of materials for building construction, as most of them exceed the proposed dose of 1 mSv·yr^−1^ in the case of their single examination. The presented simulation of the actual radiation emission demonstrates that the obtained results are lower than the dose proposed by RP112 [13] at the level of 0.5 mSv·yr^−1^. Comparing the obtained values with the data obtained for materials used in other European Union countries, it appears that they significantly exceed them with the emission of ionizing radiation.

The presented radioactivity of building materials is crucial in the current trend in the construction of zero-emission and passive facilities. The necessity to implement the provisions of the Council Directive 2013/59 EURATOM [4] and specialized institutions, such as WHO [24] and ICRP [25], have shown that the construction of residential buildings with materials containing radioactive substances requires special radiological attention, owing to the fact that their natural radiation may harm residents in the case when certain exposure limits are exceeded.

## 6. Conclusions

Radionuclide concentrations expressed by the Radioactivity Concentration Index found in building materials available in Poland indicate a low probability of increased exposure to radiation (<1 mSv·yr−1). The radioactivity of all analyzed materials, taking into account to the radiation of the reference sample, complies with national and international guidelines. The use of building materials for insulation purposes (e.g., mineral wool, gypsum boards, OSB boards, tiles) limits the radiation of basic construction materials—bricks and concrete products. Radionuclide concentrations recorded in Polish building materials are comparable to the results obtained by scientists in other countries and with the use of other research methods. Despite the presence of higher radiation emission results for some materials compared with the literature data, they are not significant for the health of users. Problems with maintaining the proper level of natural radioactivity indicators of building materials may appear in materials, which will include additives of industrial origin, such as slags or ashes. In such a case, production recipes should be used so that the final product does not exceed the maximum values of individual indicators. This applies to materials such as cements with the addition of ashes or slags, or red ceramics, where ash is used as a material that slows down and reduces shrinkage during firing. The proposed measurement method with the use of the EKO-C dosimeter may prove to be effective for determining the radioactivity concentration index in measurements performed in non-laboratory conditions. In order to correctly interpret the obtained test results, it requires calibration with standardized methodologies and requires a series of material tests to determine the appropriate threshold values and sensitivity of the method.

## Figures and Tables

**Table 1:** Average radioactivity concentration in building materials for Poland [19]. EU, European Union.

Material	Number of Samples	^226^Ra (Bq·kg^−1^)	^232^Th (Bq·kg^−1^)	^40^K (Bq·kg^−1^)	I (mSv·yr^−1^)
bricks	6	16	20	515	0.3
concrete	678	115	72	263	0.8
cement	344	73	66	353	0.7
gypsum	502	15	9	91	0.1
superficial stone—plutonic	387	78	89	1049	1.1
superficial stone—volcanic	86	160	163	1295	1.8
superficial stone—metamorphic	148	27	21	395	0.3
EU natural soil (for comparison)	23	36	34	483	0.5

**Table 2:** Radioactivity concentration index measured for bricks.

Material	I (mSv·yr^−1^)			Coefficient of Variation (%)	Measurement Expanded Uncertainty (%)
	Max	Min	Average		
construction brick	6.21	5.25	5.78	5.33	0.35
full clinker brick	10.77	10.77	11.30	2.97	0.38
hollow mould brick	11.56	9.63	10.51	5.44	0.65
classic brick	7.01	5.43	6.48	7.51	0.55
reference sample	5.41	5.39	5.40	0.11	0.24

**Table 3:** Radioactivity concentration index measured for structures and facades.

Material	I (mSv·yr^−1^)			Coefficient of Variation (%)	Measurement Expanded Uncertainty (%)
	Max	Min	Average		
ceramic block	16.64	14.89	15.76	3.26	0.60
shuttering block	13.57	13.57	13.57	0.00	0.50
concrete slab	11.82	10.51	11.03	4.00	0.50
aerated concrete	11.65	10.51	10.51	2.64	0.30
reference sample	5.41	5.39	5.40	0.11	0.24

**Table 4:** Radioactivity concentration index measured for insulating materials.

Material	I (mSv·yr^−1^)			Coefficient of Variation (%)	Measurement Expanded Uncertainty (%)
	Max	Min	Average		
classic polystyrene	4.99	3.59	4.11	9.53	0.45
graphite polystyrene	4.46	4.46	4.46	5.81	0.33
glass wool	2.71	1.92	2.36	9.59	0.26
mineral wool	3.24	2.10	2.45	2.13	0.34
reference sample	5.41	5.39	5.40	0.11	0.24

**Table 5:** Radioactivity concentration index measured for finishing plates.

Material	I (mSv·yr^−1^)			Coefficient of Variation (%)	Measurement Expanded Uncertainty (%)
	Max	Min	Average		
plywood	3.94	2.97	3.15	3.26	0.23
gypsum board	4.20	3.32	3.76	7.56	0.33
OSB board	3.32	2.62	2.89	6.34	0.21
MFP board	3.50	2.80	3.15	6.36	0.23
reference sample	5.41	5.39	5.40	0.11	0.24

**Table 6:** Radioactivity concentration index measured for ceramic and stone tiles.

Material	I (mSv·yr^−1^)			Coefficient of Variation (%)	Measurement Expanded Uncertainty (%)
	Max	Min	Average		
ceramic tiles	1.13	0.96	1.05	1.15	0.11
metamorphic stone tiles	0.82	0.13	0.48	2.93	0.19
reference sample	5.41	5.39	5.40	0.11	0.24

## Data Availability

The data presented in this study are available on request from the corresponding author.
